# Research on healing-oriented street design based on quantitative emotional electroencephalography and eye-tracking technology

**DOI:** 10.3389/fnhum.2025.1546933

**Published:** 2025-05-13

**Authors:** Huibin Shao, Ying Liu, Hongguo Ren, Zhenyu Li

**Affiliations:** ^1^International Research Center of Architecture and Emotion, Hebei University of Engineering, Handan, China; ^2^Bingtuan Xingxin Vocational and Technical College, Institute of Information Engineering, Tiemenguan, China

**Keywords:** electroencephalography (EEG), eye-tracking technology, street design, healing, quantitative emotional

## Abstract

This study seeks to evaluate how street space design impacts users’ psychological healing. In the introduction, we highlight the significance of this research area and outline our objectives. Regarding methods, we employed Electroencephalography (EEG) and eye - tracking technologies, centering on two design elements: street interface type and green ratings, and used physiological indicators to measure users’ healing responses. As for results, EEG data showed that open space interfaces reduce cognitive load while high green ratings offer positive natural stimulation, both enhancing healing effects. Eye - tracking data indicated that green ratings might influence healing more than interface type, likely due to their role in determining natural element richness. The subjective questionnaire also underlined the importance of being away and extent in street healing. In the discussion, we confirm the effectiveness of EEG and eye - tracking in assessing street space healing and present an evaluation framework blending physiological and emotional responses. Overall, this study provides an empirical basis for optimizing street space design and improving design interventions.

## 1 Introduction

### 1.1 Research background

In today’s fast-developing world, urbanization is accelerating, the pace of people’s lives is getting faster, and mental health problems are becoming more and more prominent. In 2019, the World Health Organization pointed out that nearly 1 billion people around the globe are suffering from mental disorders, with 14% of them in the adolescent group. In such a social context, the design of the street, as a transportation space connecting various functional areas in the city, not only affects the operational efficiency of the city, but is also closely linked to the daily life and mental health of residents. However, most of the current street space design is still dominated by the basic transportation function, often ignoring the healing potential contained in the street space (Romans et al., 2011). The concept of healing street design has emerged, aiming to create a space for residents to relax and enjoy themselves through natural elements and landscape layout (Li and Xu, 2020). This design not only enhances residents’ quality of life, but may also have a positive impact on their mental health, helping to alleviate negative emotions such as stress and anxiety. Previous studies on the healing properties of streets are mostly based on field surveys and use qualitative research methods, which mainly rely on subjective feelings and empirical judgments and lack scientific verification. Although this research method can provide some reference, it is difficult to accurately assess the differences in the psychological healing effect of street design on users due to its subjectivity and limitations. In addition, there are relatively few quantitative studies on people’s healing perceptions in streets, limiting researchers’ in-depth understanding and scientific knowledge of this field. Perceptual architecture, on the other hand, as an interdisciplinary field, integrates the theoretical foundations of perceptual engineering and architecture, and through the application of the principles of cognitive science and advanced signal acquisition technologies, it provides in-depth analysis of human perceptual experiences and emotional responses in architectural spaces (Ren et al., 2024). This study aims to scientifically assess the impact of street space design on the psychological healing effect of users, using electroencephalography (EEG) and eye tracking technology, focusing on two core design elements, namely, the type of street interface and the level of green ratings, and utilizing physiological metrics to quantify the users’ healing response. Through experimental design and data analysis, the study quantifies the emotional state of human beings and deeply explores the influence of different street spaces on the healing effect of human beings. The study not only establishes the validity of EEG and eye-tracking techniques in the assessment of healing in street spaces, but also proposes a scientific evaluation framework that integrates the physiological and emotional responses of users, which provides an empirical basis for the improvement and optimization of the healing design of street spaces, and strengthens the relevance and effectiveness of the design interventions.

### 1.2 EEG and related theoretical studies

As a non-invasive neurophysiological measurement technique, the application of EEG in the landscape field is mainly reflected in the assessment of environmental quality and landscape preference through visual stimuli (Kim et al., 2019). EEG is able to capture the brain’s real-time response to landscape stimuli, thus providing a scientific basis for the optimization of landscape design (Hu and Roberts, 2020; Yamane et al., 2002; Tilley et al., 2017). In the analysis of EEG signals, four main EEG bands are usually focused on: α-waves (8–13 Hz), β-waves (14–30 Hz), θ-waves (4–7 Hz) and δ-waves (0.5–4 Hz) (Lindsley, 1952). These waves reflect the pattern of neural activity of the brain in different cognitive states. α-waves are associated with a relaxed state and non-stressed wakefulness of an individual, whereas β-waves are associated with alertness, thinking, attentional focus, and heightened cognitive activity (Cooper et al., 1974; Babiloni et al., 2009). And the ratio of α-waves to β-waves (RAB: α/β) is considered to be an important indicator closely related to behavior, which can reflect the activity state of the brain during cognitive tasks. A significant increase in the ratio of α/β power during a cognitive task indicates that the brain is feeling healed (Griffiths et al., 2019). Through these indicators, researchers can gain a deeper understanding of human emotional and cognitive responses to different landscape or built environment stimuli, and thus promote the humanization and optimization of landscape design.

Furthermore, previous studies have provided insights into the functions of different brain regions in emotional and cognitive responses. For example, it has been noted in the literature that the frontal lobe is closely related to the emotional regulation and decision-making processes of individuals, and its processing of environmental stimuli is associated with emotional experience and behavioral control. The central lobe plays a key role in sensorimotor integration and cognitive control, and its activity reflects the degree to which an individual attends and responds to environmental stimuli. The temporal lobe is associated with auditory processing and memory formation, and it has an important role in processing environmental sounds and memorizing information. The parietal lobe is mainly responsible for the processing of sensory information and the modulation of spatial attention, and its activity is related to the individual’s tactile and spatial perception of the environment. The occipital lobe serves as the center of visual information processing, and its activity directly reflects the individual’s ability to process and analyze visual stimuli. These studies provide a theoretical basis for the selection of EEG detection locations in this study and help us better understand the role of different brain regions in street healing assessment (Roland and Zilles, 1998).

EEG technology has become an effective means of monitoring human emotional changes in response to stimuli from the landscape and built environment. For example, scholars measured the EEG of 30 healthy students through a field experiment in two subway stations with different comfort levels and found that participants in the uncomfortable subway station experienced more stress (Kim et al., 2020). Deng et al. (2021) experimentally studied EEG data under different indoor lighting conditions to explore the effect of light levels on work engagement and used physiological sensing technology combined with machine learning models to predict an individual’s level of work engagement under different light levels. Mir et al. (2022) investigated the effects of different types and levels of building construction noise on human emotions through EEG data and used a questionnaire to assess noise-induced annoyance and stress levels. Zhang et al. (2023) explored the effects of noise on human emotions through experiments in immersive virtual environments (IVEs), using EEG to measure participants’ brain activity, explored the effects of short-term exposure to natural environments on psychological functioning and analyzed the potential mechanisms of such effects. Lin et al. (2020) used a mobile EEG device to compare the effects of two different behaviors, walking and sitting in an urban green space, on mood changes in college students and found that walking was more helpful in reducing stress, while sitting was more conducive to attention recovery. Rounds et al. (2020) investigated the effects of architectural design features on brain activity during landmark recognition and navigation tasks in an urban environment through the use of virtual reality (VR) and EEG analysis. Olszewska-Guizzo et al. (2020) explored the effects of a specific urban green space on prefrontal α-wave brain asymmetry (FAA) to assess the potential link between the visual quality of urban green spaces on mental health outcomes. Cho et al. (2022) explored the effects of urban design and the level of environmental management on people’s positive and negative emotions in a shrinking neighborhood in Seoul, South Korea, through an EEG study conducted in a shrinking neighborhood. Imperatori et al. (2023) explored the effects of natural environmental exposure on functional connectivity within the emotional distress network through a resting-state EEG study and found that exposure to natural environments was associated with reduced deltaic functional connectivity compared to urban environments, particularly between the left insula and the left anterior cortex of the left subcingulate gyrus. In summary, EEG is currently being used to study a number of aspects of human work engagement levels, attention recovery, stress tests, and mood changes in public spaces, but not much attention has been paid to the healing aspects of evaluating street spaces.

### 1.3 Eye-tracking technology and related theoretical research

Eye-tracking technology is used in several fields to assess visual quality and provide design recommendations (Punde et al., 2017; Poole and Ball, 2006). It has been used in conjunction with questionnaires to analyze the visual quality of public spaces (Xing and Leng, 2023), to explore the effects of color attributes on visual comfort in subway spaces (Zhang et al., 2022), to study differences in people’s visual processing of green areas in residential yards (Nadezhda et al., 2022), to analyze pedestrians’ visual engagement with street edges (Simpson et al., 2022), to record the use of visual information by people with different levels of visual acuity in street environments (Yuji et al., 2020), to conduct user-centered museum assessments (Zhou et al., 2023), and studying visual landscape preferences in urban environments (Fadda et al., 2023). These applications demonstrate the advantages of eye-tracking technology in objectively recording visual attention and perceptual processes, overcoming the limitations of traditional subjective evaluations.

### 1.4 Synchronized detection of EEG-eye-tracking technology and related theoretical studies

The combination of EEG and eye-tracking technology allows researchers to quantify an individual’s physiological response to the environment and to study the interaction of brain activity with visual attention. This technique also improves the quality of the EEG signal and removes artifacts through eye movement data. Studies have shown differences in visual preferences and physiological responses to plant features in individuals from different backgrounds (Bianconi et al., 2019). Another study explored visual preferences for indoor spaces among participants of different backgrounds and genders (Ding et al., 2022). Still another study analyzed the effect of the number of landmarks in a virtual environment on the cognitive load of navigators (Cheng et al., 2023). Simultaneous monitoring of eye movements and EEG revealed pedestrian avoidance behaviors in subway public spaces in subway stations, providing theoretical support for environmental design (Tang et al., 2023).

In this study, we assessed the effect of street scenes on human healing through EEG and eye-tracking techniques, providing neurophysiological evidence for urban planning and design.

## 2 Materials and methods

This study relies on EEG and eye-tracking technology to capture physiological signals from the human body in order to deeply explore individuals’ healing feelings in different street spaces, aiming to reveal the potential impact of different street spaces on people’s healing effect.

### 2.1 Experimental equipment

In this study, EEG activity was recorded through a 64-channel lead-count electrode cap, of which 32 electrodes were selected for data acquisition at a sampling rate of 1,024 Hz, using the SAGA system for EEG wave data acquisition. The selected electrodes covered the major regions of the brain, including occipital lobe (O1, Oz, O2, and Poz), frontal lobe (FP1, FP2, FPz, F7, F3, Fz, F4, F8, FC1, FC2, FC5, and FC6), temporal lobe (T7 and T8), parietal lobe (P3, P4, P7, P8, Pz, CP1, CP2, CP5, and CP6), central lobe (C3, C4, and Cz), and M1 and M2 regions of the motor cortex. For eye tracking, the aSee Pro remote eye tracking system was used, which allowed participants to record their eye movement data at 140 Hz without wearing any equipment. During the experiment, participants were located in front of a 21-inch LCD monitor and maintained a viewing distance of approximately 60 cm from the monitor. To ensure consistent lighting conditions in the experimental environment, blackout curtains were used in the laboratory to isolate external light sources, and indoor lighting was provided by incandescent lamps on top. The data analysis software, aSee Studio, was installed on a PC equipped with an Nvidia GTX1060 graphics card, which has a resolution of 1,920 × 1,080 and runs the 64-bit Windows 10 Pro operating system.

### 2.2 Process

**Figure 1:**
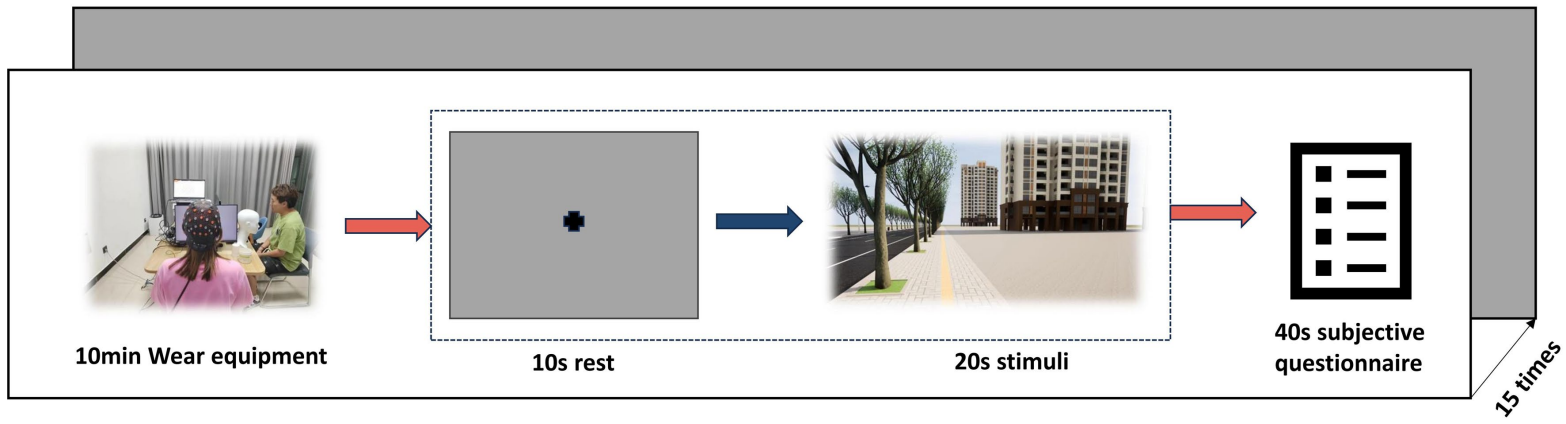
Process.

The experimental design was succinctly divided into three consecutive phases: first, participants adapted to the environment with their eyes closed and a baseline EEG was recorded; next, EEG and eye movement data were synchronized and captured while they viewed 15 sets of scene images; and, finally, participants made a subjective assessment of their comfort with the landscape for each set of images, with the entire experiment taking approximately 17 min and 30 s (as shown in Figure 1).

### 2.3 Participants

In order to ensure the scientific validity of the experimental process and the accuracy of the data, More Power 6.0.4 software was used to calculate the required sample size, and it was calculated that at least 32 participants were needed for the study.

Strict screening criteria were set for this study to ensure the accuracy of the EEG and eye movement data and to exclude individual and environmental variables that might affect the results. Participants were required to have the ability to think and express themselves clearly, have a visual acuity of 1.0 or higher, and have no visual problems such as color blindness or color weakness. All the participants have been informed and signed informed consent before the experiments, and they can withdraw at any time if he/she feels any discomfort. Eventually, 34 undergraduate and master’s students (15 males and 19 females) between the ages of 20 and 30 were selected to participate in the experiment, all of whom were informed of the study objectives and procedures prior to the experiment.

### 2.4 Stimulus and elemental design

**Figure 2:**
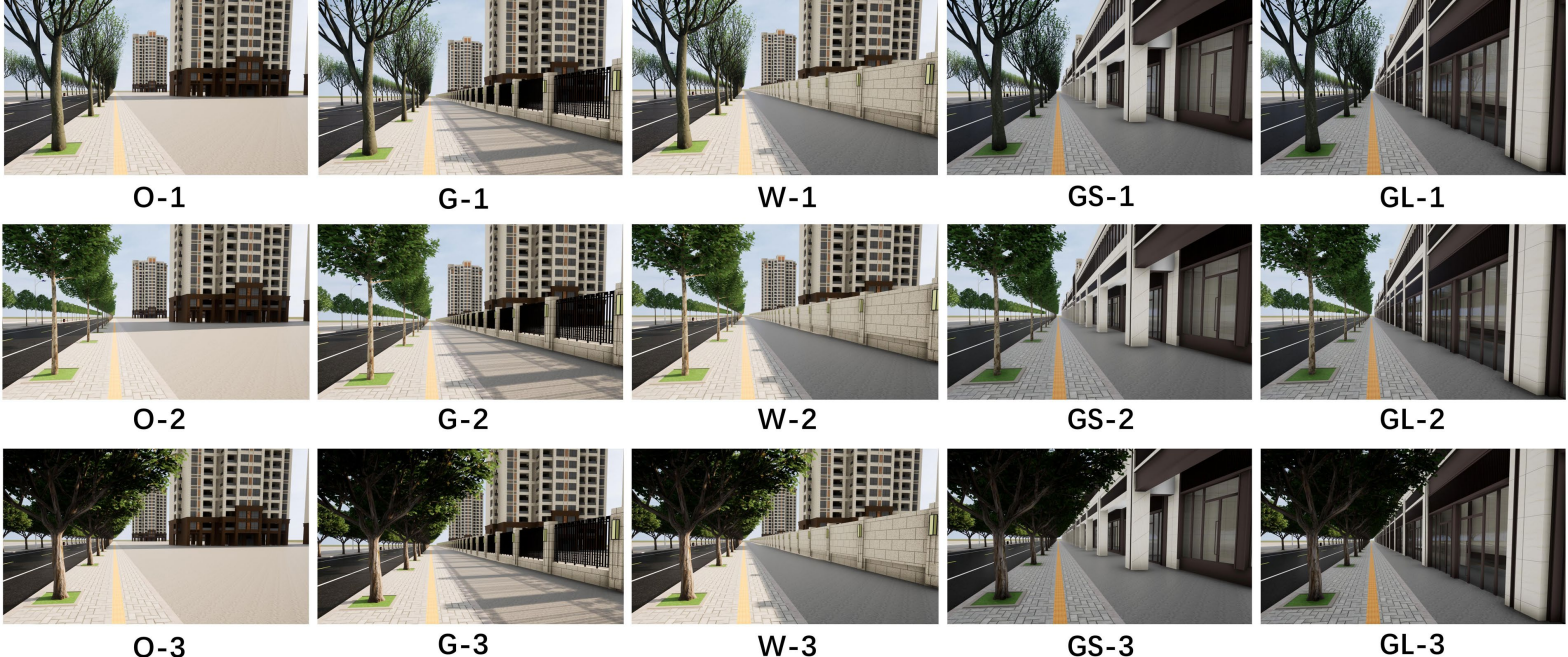
15 scenario models.

In a study by Xu et al. (2019) they explored the healing properties of urban green space landscapes, especially the effect of green rating on people’s psychological and physiological health. Green rating refers to the proportion of green plants within a person’s field of vision, and this indicator is considered to be an important parameter for assessing the healing properties of urban green spaces. As mentioned in the study, green rating is affected by a variety of factors, including object factors such as the type, quantity and layout of vegetation, as well as subject factors such as the observer’s position and perspective. In terms of the design of this experiment, the researcher chose the crown width of street trees as the independent variable, while other factors that may affect the green rating were used as control variables. The green rating was categorized into three levels in the experiment: low (less than 15%), medium (15 to 25%), and high (25% and above). In addition, the study considered the types of street interfaces, including open interfaces, grille interfaces, solid wall interfaces, glass interfaces, and gray space interfaces, which may have different effects on the psychological healing effect on people. The three independent variables of green rating and the five independent variables of street interfaces were fully ranked to obtain 15 scenario models (as shown in Figure 2) (O open interfaces, G grille interfaces, W solid wall interfaces, GL glass interfaces, GS gray space interfaces, 1 low green rating, 2 medium green rating, and 3 high green rating).

To ensure experimental consistency and user experience, the street scenes in the study were designed to maintain a consistent viewing height (1.70 m) and high resolution photographs (2,560 × 1,440) were used to present the experimental stimuli. This approach better simulates people’s visual experience in real street environments and takes into account that photographs have been shown to positively influence brain and autonomic nervous system activity (Yamashita et al., 2021).

### 2.5 Data indicator extraction

This study comprehensively evaluated 34 sets of indicators based on participants’ perceptions and physiological responses, which were categorized into three main categories: EEG, eye-tracking data, and subjective questionnaire scores. The categories of indicators and their scientific significance are listed below.

#### 2.5.1 EEG data indicators

In this study, participants’ electroencephalogram (EEG) signals were acquired through 32 channels using a sampling frequency of 1,024 Hz and processed by a 16-bit analog-to-digital converter (ADC). The signals are stored in a frequency passband of 0.003–150 Hz to ensure that a broad spectrum of EEG activity can be captured. A SAGA acquisition and analysis system, which is known for its portability and high data quality, was utilized to record raw EEG data in real time. In the signal preprocessing stage, a 1–50 Hz bandpass filter (BPF) was used to remove extraneous frequency components, and the EEG signals were analyzed spectrally by means of the fast Fourier transform (FFT) method, an efficient algorithm capable of converting time-domain signals into frequency-domain signals, revealing the frequency components of the signals. In addition, the characterization of EEG channels is performed by calculating the ratio between alpha band (8–12 Hz) and beta band (12–30 Hz) power as shown in Equation 1, and this ratio can reflect the different functional states of the brain.


(1) $$RAB=\frac{Power\;\alpha }{Power\;\beta }$$

#### 2.5.2 Eye-tracking data indicators

The data indicators obtained from the eye-tracking experiment, including gaze point, gaze duration, eye hopping, and pupil diameter change, as shown in the table, can reveal the distribution of attention and cognitive activities of individuals when processing visual information. The analysis of these metrics contributes to an in-depth understanding of participants’ attentional patterns when observing visual stimuli, providing an empirical basis for user experience research.

Two data indicators, gaze duration and area of interest AOI, were used in this study. One of the key metrics is gaze duration, which is the average of the duration of each gaze a participant makes during the observation of a visual stimulus. This metric can reflect the depth and complexity of visual processing, and usually, a longer gaze duration implies that the participant may have been observing a specific region in more detail or encountered some cognitive difficulties (Minoru et al., 2023). The area of interest AOI, on the other hand, is a specific region of visual stimuli identified by the researcher as a key area for probing the distribution of visual attention. By setting the area of interest (AOI), researchers are able to quantify and evaluate eye movement parameters such as participants’ gaze position and duration on specific visual content, and thus infer the level of attentional focus and the depth of visual information processing.

#### 2.5.3 Subjective questionnaire indicators

The Perceived Restorative Scale (PRS) is a commonly used tool for assessing the restorative effects of an environment, which consists of four main dimensions: being away, fascination, extent, and compatibility. Being away refers to whether the environment is able to provide a scenario that is very different from everyday life, thus helping people to escape from stress; fascination focuses on the aesthetics of the environment and its ability to divert attention and relieve fatigue; extent explores the richness and coherence of the environment’s content and its ability to attract the user’s attention and provide opportunities for rest; and compatibility measures the extent to which the environment’s activities match the user’s interests and behavioral needs, as well as whether they can stimulate empathy. The scale is rated on a Richter scale ranging from 1 (least compatible) to 7 (most compatible), and the opposite is true for the extent score. Data analyses typically draw conclusions based on the mean of the total score of the Perceived Recovery Evaluation, and the mean and standard deviation of the dimensions.

#### 2.5.4 Data analysis methods

In this empirical study, the researchers included a total of 34 participants and collected a multimodal dataset including EEG data, subjective rating questionnaires, and eye-tracking data. The data collection rate for all participants exceeded 95%, ensuring the credibility and validity of the dataset. The researchers implemented a standardized analysis process to obtain the basic data required for the experiment through accurate data collection techniques. A variability analysis was executed on the data and statistical methods were utilized to assess whether significant differences existed between the data under different experimental conditions. In addition, in order to reveal potential associations between variables, the researcher performed correlation analysis on the data. Based on the combined results of these analyses, the conclusions of the experiment were drawn, and these findings are thoroughly explored and explained in the discussion chapter.

## 3 Result

### 3.1 Analysis of data variability

In this study, the data sets were first analyzed for data variability to investigate whether there were significant differences in the physiological and cognitive data of the subjects between the two groups of different scenarios produced by changes in the independent variables.

#### 3.1.1 EEG analysis of variance

The green ratings and street interface was used as the independent variable and the EEG data as the dependent variable. In this paper, Brain ERS is used to process the raw data, and the data is first processed by Min-Max Scaling as shown in Equation 2, which helps to eliminate the influence of the magnitude between different features, making the model training more efficient and stable. Where x is the original data point, $x_{\min}$ is the minimum value in the dataset, $x_{\max}$ is the maximum value in the dataset, and $X_{\mathrm{norm}}$ is the normalized data point.


(2) $$X_{\mathrm{norm}}=\frac{x-x_{\min}}{x_{\max}-x_{\min}}$$

The data of 34 participants on the five brain regions of 15 groups of scenes were also analyzed for variability by IBM SPSS Statistics 27. The changes in the data of the 15 groups of different scenarios on different participants were statistically significant only if the *p*-value was ≤the significance level of 0.05, proving that the changes in the independent variables had a significant effect on the physiological data of the participants.

In this paper, a multifactorial ANOVA was used for the preliminary analysis of the data as shown (as shown in Table 1). The significance (*p*-value) of the street interface on all five brain regions was <0.05, proving that the change in the street interface had an effect on the physiological data of the participants. The significance (p-value) of green rating on all five brain regions was also <0.05, again proving that the change in green rating had an effect on the physiological data of the participants. However, the significance (p-value) of the interaction between street interface and green rating on all five brain regions was greater than 0.05, indicating that the interaction of green rating on street interface did not show a significant difference on the physiological data of the participants.

**Table 1:** Multifactor ANOVA for preliminary analysis of data.

Origin	Implicit variable	Type III sum of squares	Degrees of freedom	Equal square	*F*	Significance
Street interface	Frontal lobe	14.027	4	3.507	259.775	<0.001
	Central lobe	20.121	4	5.030	201.304	<0.001
	Temporal lobe	19.986	4	4.996	286.854	<0.001
	Parietal lobe	19.971	4	4.993	272.406	<0.001
	Occipital lobe	18.813	4	4.703	201.719	<0.001
Green rating	Frontal lobe	3.413	2	1.707	126.417	<0.001
	Central lobe	5.017	2	2.508	100.387	<0.001
	Temporal lobe	4.661	2	2.330	133.787	<0.001
	Parietal lobe	5.416	2	2.708	147.749	<0.001
	Occipital lobe	5.480	2	2.740	117.506	<0.001
Street interface * Green rating	Frontal lobe	0.049	8	0.006	0.453	0.889
	Central lobe	0.215	8	0.027	1.075	0.379
	Temporal lobe	0.146	8	0.018	1.049	0.398
	Parietal lobe	0.124	8	0.016	0.848	0.561
	Occipital lobe	0.114	8	0.014	0.613	0.767

The data were then compared *post hoc*, and according to the *post hoc* comparison S-N-K table, it was learned that the lowest mean square (M) of the independent variable street interface among the five brain regions was the solid wall interface. The next lowest was the fence interface. The mean squares (M) of the glass and gray space interfaces differed somewhat across brain regions, with the mean squares (M) of the gray space interface being greater than that of the glass interface in the frontal and central lobes, and the *p*-values of within-group comparisons between the gray space interface and the glass interface in the temporal, parietal, and occipital lobes being greater than 0.05, which is not a statistically significant difference. The mean square (M) was largest for the empty space interface. The lowest mean square (M) for the independent variable greenness was low greenness in all five brain regions, followed by medium greenness, and the best was high greenness, but the *p*-values for within-group comparisons of medium greenness and high greenness were >0.05, which was not statistically significantly different.

In order to explore the changes in RAB values of the EEG data for the 15 sets of scenarios, a box-and-line plot of the changes in the average RAB values of the five brain regions was plotted using Origin2022 software. The horizontal axis represents the 15 scene serial numbers and the vertical axis represents the RAB values.

**Figure 3:**
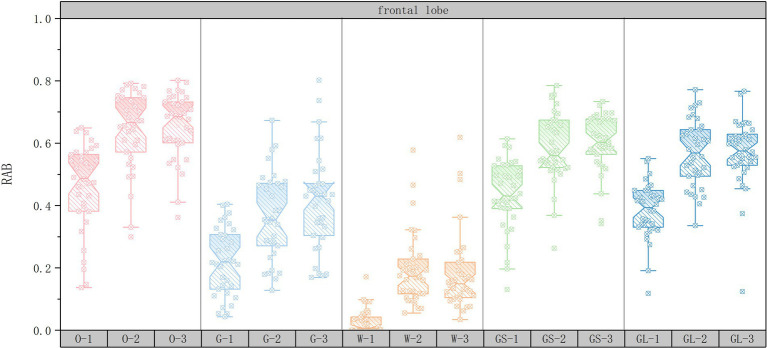
Box plots of changes in frontal lobe mean RAB values.

As shown in Figure 3, in the frontal region, the RAB value of the open space interface was significantly higher than that of the other interfaces (70.59% of the subjects exhibited the highest RAB value), suggesting that the sense of order and controllability of the open space may have significantly enhanced the healing effect by either decreasing the cognitive load or enhancing the ability of attentional recovery. In addition, the combination of open space interface with medium green ratings (35.29% of subjects with the highest RAB values) further suggests that moderate natural elements combined with open space layout may further enhance the healing effect by optimizing visual stimulation and cognitive regulation. In contrast, although the high green ratings scene had some enhancement of healing, its RAB value was not as significant as that of the open space interface (52.94% of the subjects had a higher RAB value than the medium green ratings), which may indicate that the enhancement of healing in the frontal lobe area relies more on the optimization of the spatial layout than solely on the abundance of natural elements.

**Figure 4:**
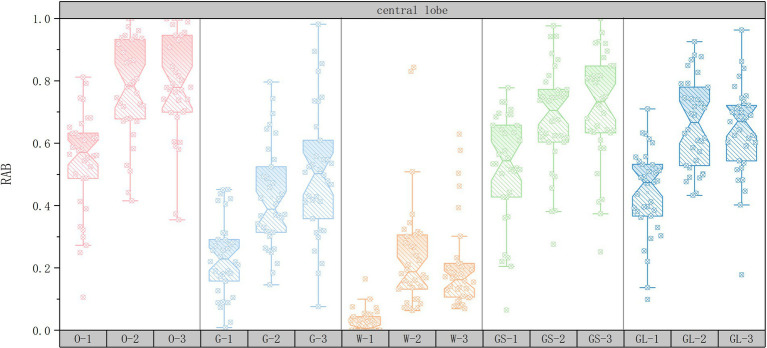
Box plots of changes in central lobe mean RAB values.

As shown in Figure 4, in the central leaf area, the RAB values of the green high-vis scene were significantly higher than those of the medium-green ratings (58.82% of subjects had higher RAB values), suggesting that visual stimulation by natural elements may have directly enhanced the healing effect through emotional calming or stress relief. Although the RAB value of the open space interface was also higher (55.88% of subjects had the highest RAB value), the enhancement of its healing effect may have relied more on synergizing with the high green ratings (29.41% of subjects had the highest RAB value in the combination of the open space interface and the high green ratings). This suggests that the healing enhancement of the central leafy area relies more on the direct perception of natural elements than on the optimization of the spatial layout.

**Figure 5:**
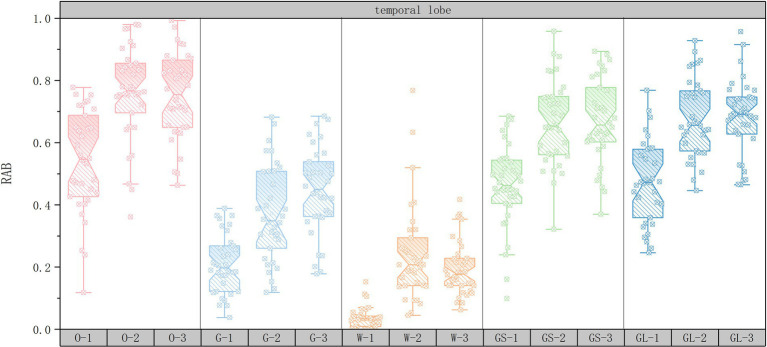
Box plots of changes in temporal lobe mean RAB values.

As shown in Figure 5, in the temporal lobe region, the RAB value of the open space interface was significantly higher than that of the other interfaces (58.82% of the subjects showed the highest RAB value), suggesting that the sense of order and controllability of open space may have significantly enhanced the healing effect by reducing the cognitive load or enhancing the ability of attentional recovery. In addition, the glass interface had higher RAB values than the gray space (55.88% of subjects), suggesting that visual permeability may further enhance healing by optimizing the perceptual experience. The RAB values of the high green ratings scene were higher than those of the medium green ratings (52.94% of subjects), suggesting that visual stimulation by natural elements has an enhancement effect on healing, but its effect was not as significant as that of the open space interface. The combination of open space interface and medium green ratings (35.29% of subjects with the highest RAB values) further suggests that moderate natural elements combined with open space layout may have further enhanced the healing effect by optimizing visual stimulation and cognitive modulation.

**Figure 6:**
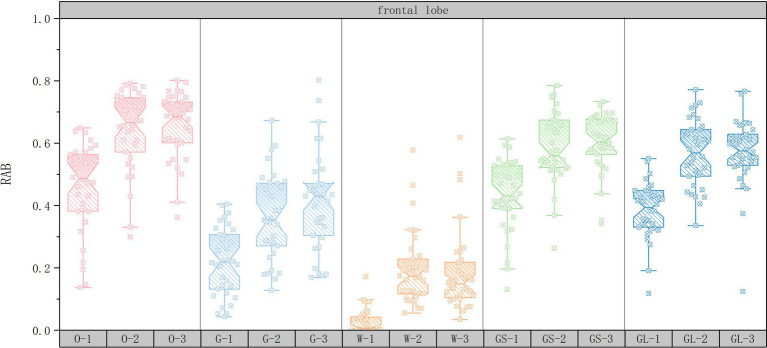
Box plots of changes in parietal lobe mean RAB values.

As shown in Figure 6, in the parietal area, the RAB value of the open space interface was the highest (64.71% of subjects), indicating that the sense of order and controllability of the open space had a significant effect on the healing enhancement. The RAB value of the grey space interface was higher than that of the glass interface (52.94% of subjects), suggesting that the sense of hierarchy and transition in grey space may further optimize the perceptual experience by blurring the boundaries and decreasing the visual pressure, thus enhancing the healing effect. The RAB value of the high green ratings scene was higher than that of the medium green ratings (50% of subjects), indicating that the visual stimulation of natural elements has a certain enhancement effect on healing, but its effect is not as significant as that of the open space interface and the gray space interface. The combination of open space interface and medium green ratings (32.35% of subjects with the highest RAB values) suggests that moderate natural elements combined with open space layout may have further enhanced the healing effect by optimizing visual stimulation and cognitive modulation.

**Figure 7:**
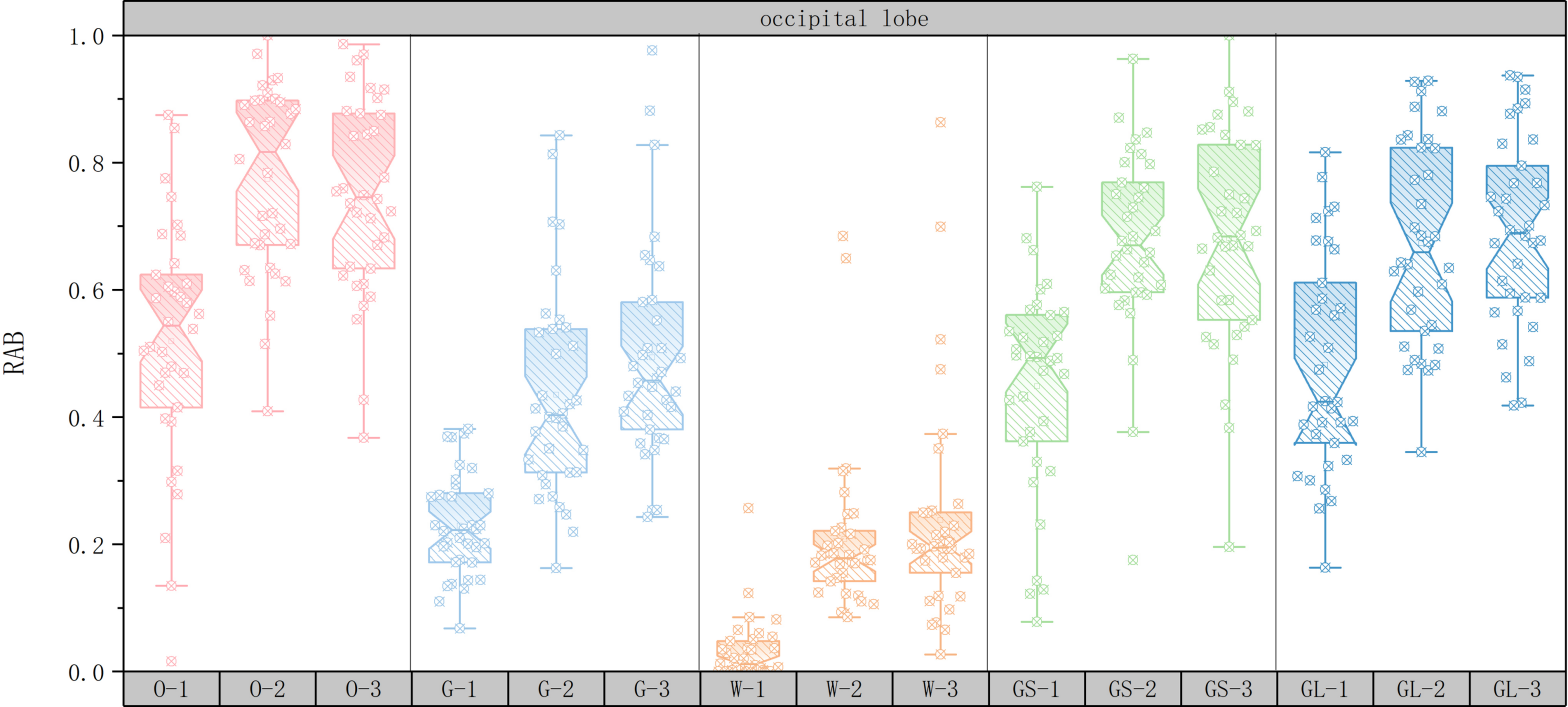
Box plots of changes in occipital lobe mean RAB values.

As shown in Figure 7, in the occipital lobe area, the RAB value of the high green ratings scene was significantly higher than that of the medium green ratings (61.76% of subjects), suggesting that visual stimulation by natural elements may directly enhance the healing effect through emotional calming or stress relief. The glass interface had higher RAB values than grey space (52.94% of subjects), suggesting that visual permeability may further enhance healing by optimizing perceptual experience. The RAB value of the open space interface was higher (47.05% of subjects), but its effect was not as significant as that of high green ratings, suggesting that the enhancement of healing in the occipital lobe area relies more on the direct perception of natural elements than on the optimization of spatial layout. The combination of open space interface and medium green ratings (38.23% of subjects with the highest RAB value) further supports the enhancement of healing by the synergistic effect of natural elements and spatial layout.

#### 3.1.2 Analysis of variance of subjective questionnaires

In this paper, the raw data of the subjective questionnaire was analyzed for differences in the data of 34 participants on the five brain regions of the 15 sets of scenarios by IBM SPSS Statistics 27. As mentioned above, the data are statistically significantly different only if the *p*-value is ≤0.05.

In this paper, a multifactor ANOVA was used for the preliminary analysis of the data (as shown in Table 2). The significance (*p*-value) of street interface on all four dimensions of the perceptual recovery scale was <0.05, proving that the street interface had an impact on the subjective evaluation of the subjects. The significance (*p*-value) of green rating on all four dimensions of the Perceptual Recovery Scale was <0.05, again proving that the green rating had an effect on the subjective evaluation of the subjects. The significance (*p*-value) of the interaction of street interface and green rating on being away, fascination, and extent in the Intuitive Recovery Scale were all >0.05, proving that the interaction of street interface and green rating did not have a statistically significant effect on these three dimensions, but the interaction of street interface and green rating on compatibility had a significance (*p*-value) <0.05, there is a significant effect.

**Table 2:** Multifactor ANOVA for preliminary analysis of data.

Origin	Implicit variable	Type III sum of squares	Degrees of freedom	Equal square	*F*	Significance
Street interface	Being away	358.724	4	89.681	129.537	<0.001
	Fascination	232.378	4	58.095	83.386	<0.001
	Extent	1154.365	4	288.591	832.746	<0.001
	Compatibility	131.916	4	32.979	22.746	<0.001
Green rating	Being away	93.608	2	46.804	67.604	<0.001
	Fascination	33.895	2	16.947	24.325	<0.001
	Extent	37.326	2	18.613	53.710	<0.001
	Compatibility	78.576	2	39.288	26.3825	<0.001
Street interface * Green rating	Being away	1.218	8	0.152	0.220	0.987
	Fascination	10.328	8	1.291	1.853	0.065
	Extent	1.906	8	0.238	0.687	0.703
	Compatibility	25.624	8	3.203	2.187	0.027

The data were then compared *post hoc*, and according to the *post hoc* S-N-K table, the mean squares (M) of the independent variable street interface in the being away dimension and extent dimension were open space interface > gray space interface > glass interface > fence interface > solid wall interface. The largest mean square (M) in the fascination dimension and the compatibility dimension was for the gray space interface, followed by the glass interface and the fence interface, but the within-group comparison *p*-value (*p* = 0.147) for both in the compatibility dimension was >0.05, which was not statistically significant. The lowest was the open space interface and solid wall interface, both of which had *p*-values > 0.05 for within-group comparisons in both dimensions and were not statistically significant. The largest mean square (M) for the independent variable green ratings among the four dimensions was high green ratings, followed by medium green ratings and the lowest was low green ratings.

Subjective evaluation scoring histograms were plotted using Origin2022 software to discuss the changes in subjective evaluations for different street scenarios. The horizontal axis represents the 15 scene serial numbers and the vertical axis represents the PRS subjective questionnaire scores.

**Figure 8:**
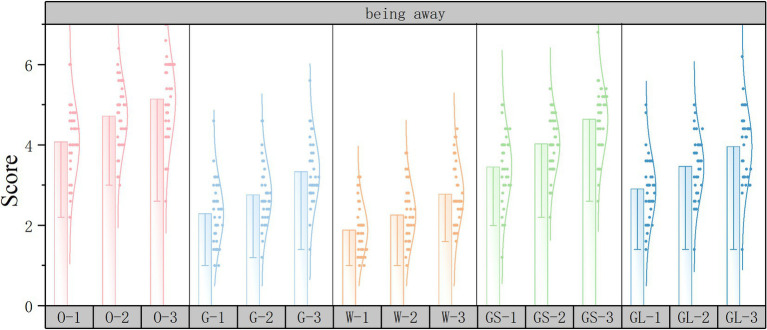
Being away subjective evaluation scoring histograms.

As shown in Figure 8, in the being away dimension, the top three scores in the independent variable street interface are open space interface, gray space interface, and glass interface, and the lowest score is solid wall interface. The independent variable green ratings had the highest score for high green ratings and the lowest score for low green ratings. This is similar to the results of the EEG data analysis. Being away itself refers to whether the environment can provide a scene that is very different from the daily life, thus helping people to escape from the stress, so being away is a dimension that needs to be emphasized in the healing street.

**Figure 9:**
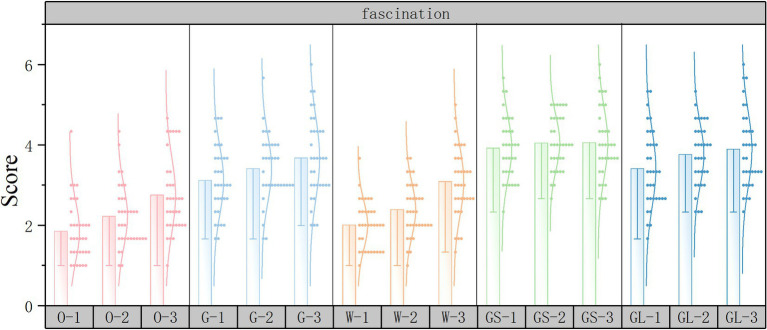
Fascination subjective evaluation scoring histograms.

As shown in Figure 9, in the fascination dimension, the top three scores in the independent variable street interface are gray space interface, glass interface, and fence interface, and the lowest score is in the open space interface. The independent variable green ratings has the highest score in high green ratings and the lowest score in low green ratings. The result of this score may lie in the fact that the open space interface and the low green ratings have few elements and simple composition, so they lack aesthetics and attractiveness.

**Figure 10:**
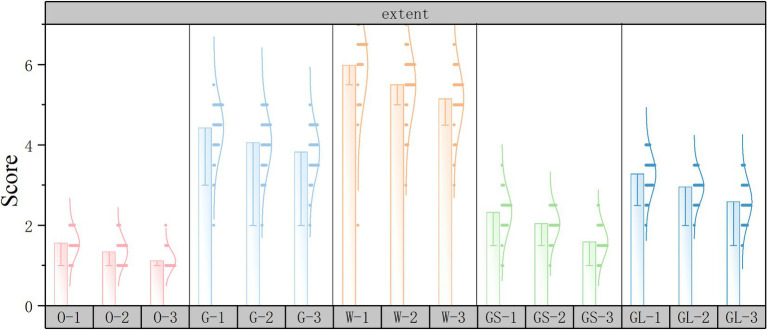
Extent subjective evaluation scoring histograms.

As shown in Figure 10, in the extent dimension, the lower the score the higher the extent and vice versa. The top three scores in the independent variable street interface are open space interface, gray space interface, and glass interface, and the lowest score is solid wall interface. The independent variable green ratings has the highest score for high green ratings and the lowest score for low green ratings.

**Figure 11:**
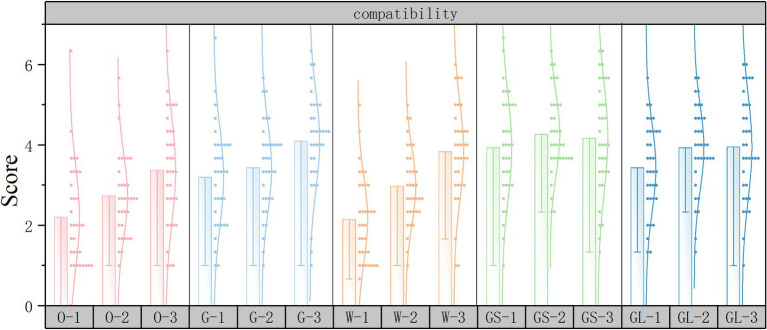
Compatibility subjective evaluation scoring histograms.

As shown in Figure 11, in the compatibility dimension, the top three scores in the independent variable street interface were gray space interface, glass interface, and fence interface, and the lowest score was solid wall interface. The independent variable green ratings has the highest score for high green ratings and the lowest score for low green ratings.

### 3.2 Data correlation analysis

Spearman’s correlation test was performed on the EEG data and subjective questionnaire data using Python 3.12, PyCharm2024.1.2 to derive the correlation coefficient $\rho_{s}$ . The values of the correlation coefficient $\rho_{s}$ ranged from −1 to +1, where values close to ±1 indicated very strong correlation, 0.6 to 0.8 (or −0.6 to −0.8) indicated strong correlation, 0.4 to 0.6 (or −0.4 to −0.6) indicates moderate correlation, 0.2 to 0.4 (or −0.2 to −0.4) indicates weak correlation, and close to 0 indicates very weak or no correlation (Spearman, 1904). And the heat map of correlation was plotted as follows, purple represents positive correlation and green represents negative correlation, and the darker the color represents stronger correlation and vice versa, weaker correlation.

**Figure 12:**
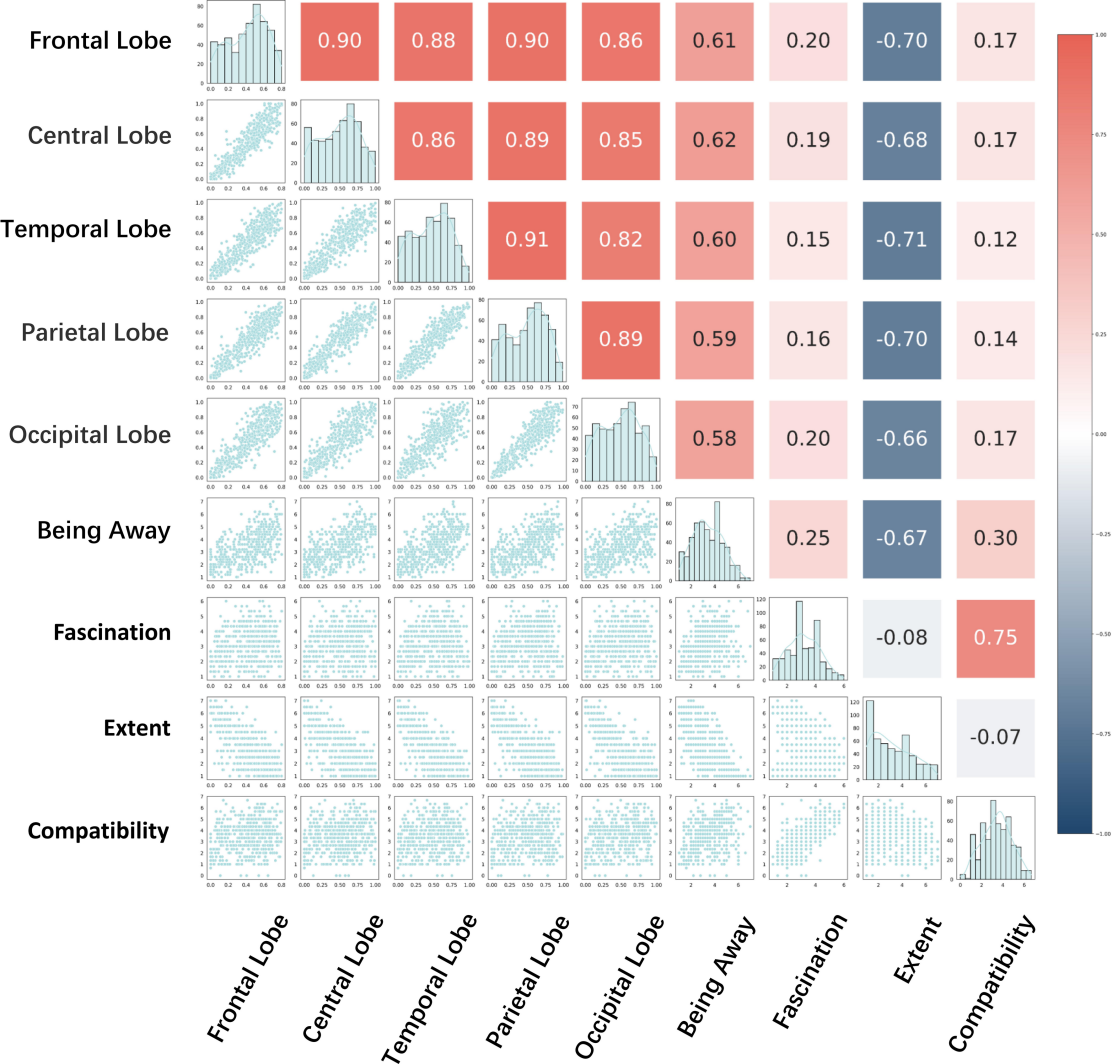
Correlation analysis.

The results of the correlation analysis highlight key associations between different physiological indicators, which provide a solid scientific basis for revealing how individuals intrinsically respond to external stimuli (as shown in Figure 12). The significant positive correlation of $\rho_{s}$ greater than 0.8 among the five brain regions indicates that the responses of the five brain regions are highly synchronized when individuals face visual stimuli. Among the four PRS dimensions $\rho_{s}$ = −0.67 was strongly negatively correlated between being away and extent, $\rho_{s}$ = 0.75 was strongly correlated between fascination and compatibility, and being away and extent were weakly correlated or very weakly correlated with each other as well as with fascination and compatibility. Being away, extent, and fascination and compatibility were weakly or very weakly correlated with each other. The five brain regions were strongly or moderately correlated with being away, strongly negatively correlated with extent, and very weakly or weakly correlated with fascination and compatibility, which suggests that the street healing effects of the PRS should be primarily considered in questions exploring the street interface and green rating as the two sets of independent variables. Being away with the extent dimension.

### 3.3 Eye tracking technology data analysis

In order to deeply investigate which group of independent variables, street interface or green ratings, plays a greater role in influencing the healing effect on subjects, 15 scenes were divided into Gridded AOIs using the eye movement eSeeStudio software, and heat maps of the total gaze duration of Gridded AOIs were plotted.

**Figure 13:**
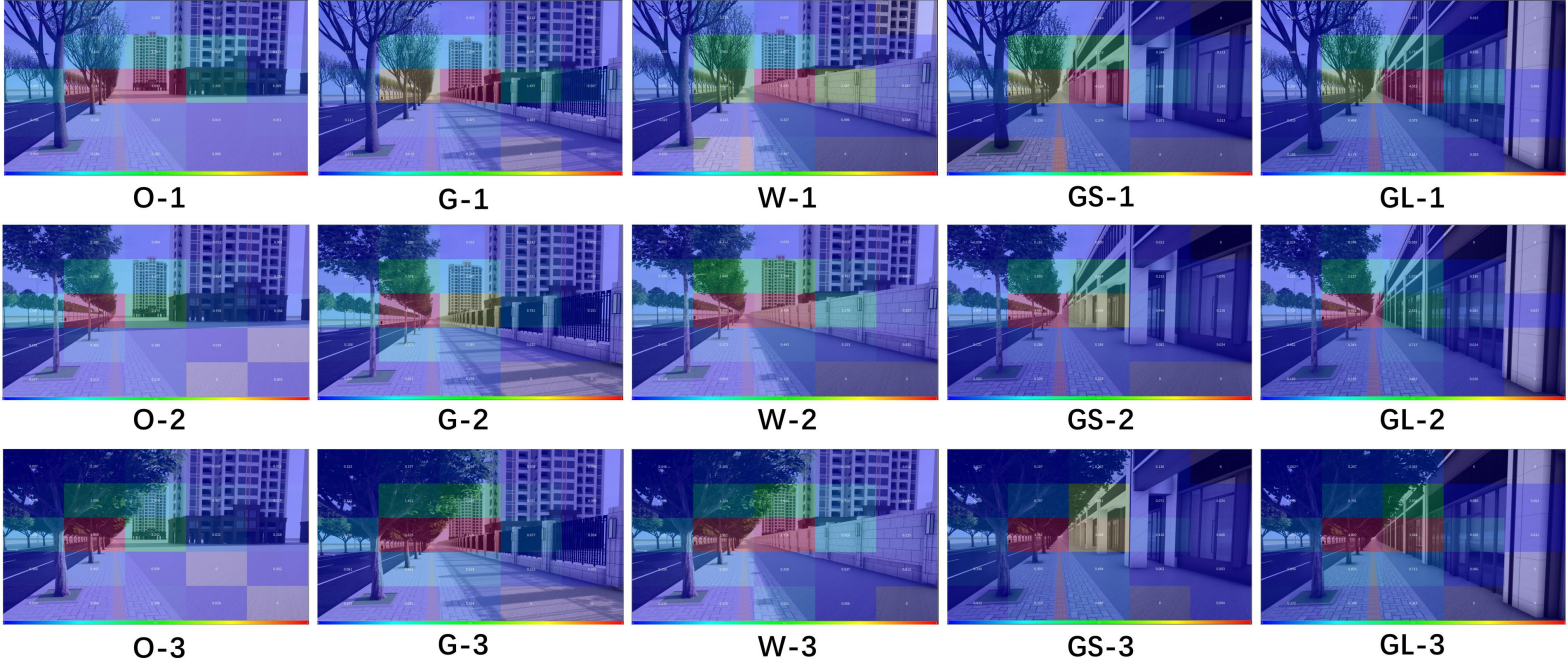
The heat map of total gaze duration of Gridded AOI.

As shown in Figure 13, in six of the 15 sets of scenes, the longest region of total gaze duration appeared in the street interface region, and five of them were scenes with the combination of low-green-vision rate and street interface. The previous analysis of the EEG data shows that the healing effect of low-green-vision is much lower than that of medium-green-vision and high-green-vision, and in the Gridded AOI heat map of total gaze duration, the longest region of the total gaze duration in nine out of the 10 groups of scenes that consisted of a combination of medium-green-vision or high-green-vision and the street interface appeared in the region of the green-vision, which proves that the subjects were more attention.

In summary, the role of the independent variable, green ratings, in influencing the subject’s healing effect from the perspective of eye-movement data gaze duration may be greater relative to the street interface.

### 3.4 Comparison with previous work

This study highlights the following innovations by comparing with existing studies (as shown in Table 3): while existing studies mostly rely on subjective questionnaires or field surveys and lack quantitative analysis and physiological data support, this study employs electroencephalography (EEG) and eye tracking techniques in conjunction with subjective questionnaires to provide scientific multimodal data support. Existing studies usually rely on a single subjective evaluation or limited physiological signals, whereas the present study uses EEG and eye tracking technology to synchronize the collection of EEG activity and visual attention data to form a comprehensive assessment framework. Existing studies focus on subjective feelings or simple physiological indicators, whereas the present study provides a more refined analysis by quantifying EEG bands (alpha/beta ratio) and eye tracking indicators (gaze duration, area of interest), combined with the four dimensions of the subjective questionnaire. While most of the existing studies remain in qualitative descriptions, this study reveals the significant effects of street interface type and green vision rate on the healing effect through experimental data analysis and suggests an optimized design. Existing studies lack scientific validation and systematic analysis, while this study fills the gap of integrating EEG and eye movement data in street healing research, providing an important reference for the theory and practice of healing street design.

**Table 3:** Comparison of this study with existing studies.

Comparative dimension	Existing studies	This study
Research methodology	Mostly based on subjective questionnaires or field surveys, lacking quantitative analysis and physiological data support	Electroencephalography (EEG) and eye-tracking techniques were used in conjunction with subjective questionnaires to provide multimodal data support
Data acquisition methods	Reliance on a single subjective assessment or limited physiologic signals (e.g., heart rate, skin conductance)	A multidimensional assessment framework was developed using EEG and eye-tracking technology to synchronize the collection of EEG activity and visual attention data, combined with subjective questionnaires
Analyzing indicators	The main focus is on subjective feelings (e.g., comfort, satisfaction) or simple physiological indicators (e.g., stress levels)	Quantitative EEG bands (alpha/beta ratio) and eye tracking metrics (gaze duration, area of interest) were combined with the four dimensions of the subjective questionnaire (being away, fascination, extent, compatibility)
Findings	Most of them remain in qualitative descriptions and lack in-depth exploration of the mechanisms affecting street healing	Experimental data analysis reveals the significant influence of street interface type and green visibility on the healing effect, and suggests recommendations for optimizing the design
Research contribution	Provides an initial reference for street design, but lacks scientific validation and systematic analysis	It fills the gap of integrating EEG and eye movement data in street healing research and provides empirical support and scientific methodology for street design

## 4 Discussion

### 4.1 Mechanisms of street interface and green ratings on healing effects

#### 4.1.1 The role of the street interface

The type of street interface has a significant effect on the psychological healing effect of people. The open space interface showed the best healing properties in all five brain regions, probably because the open space interface is relatively simple and open, which can effectively reduce the cognitive load of the individual and thus bring relaxation and comfort. The healing effect of the solid wall and fence interfaces was significantly weaker than that of the other interfaces, probably because they were more closed and monotonous, which could easily lead to a sense of depression or a lack of visual stimulation. The healing effects of the glass interface and the gray space interface differed somewhat in different brain regions, but the overall difference was not significant, which might be related to their transparency and spatial sense. The glass interface can provide a transparent view, while the gray space interface might have a certain degree of both open and sheltered characteristics, but the influence of this characteristic on the healing effect was not consistent across different brain regions.

#### 4.1.2 The role of green ratings

Green ratings acuity has a significant effect on street healing. High and medium greenery showed better healing effects in most brain regions than low greenery, suggesting that the abundance of natural elements has a positive effect on psychological healing. The abundance of greenery in the high-green-vision scene provides positive visual stimuli, which helps to alleviate stress and anxiety, and enhances the individual’s sense of pleasure and relaxation. It is noteworthy that in temporal lobe areas, the healing properties of medium greenery even exceeded those of high greenery, which may imply that moderate greenery stimulation is sufficient to produce significant healing effects in specific brain areas, while excessive greenery may not bring additional gains.

#### 4.1.3 Interaction between street interfaces and green ratings

Although street interface and green ratings each had a significant effect on the healing effect, the interaction between them was not statistically significant. This means that under the experimental conditions of the present study, changes in street interface type and green ratings levels had relatively independent effects on subjects’ physiological data and subjective evaluations, and the two did not show significant synergistic or antagonistic effects. However, the scenes with the combination of open space interface and medium green ratings showed the best healing in four brain regions, and the scenes with the combination of open space interface and high green ratings showed the best healing in one brain region, which suggests that some specific combinations may produce superior healing effects, but this combination effect did not reach a statistically significant level in the whole, and it may need to be further investigated by enlarging the sample size or optimizing the experimental design.

### 4.2 Consistency and complementarity of results from different data sources

#### 4.2.1 Consistency of EEG data with subjective questionnaires

The results of the EEG data analysis showed that the healing effect of the open space interface and the street scenes in the high and medium green ratings conditions was better for the subjects, which is consistent with the scores of the being away and the extended dimensions in the subjective questionnaire. In the subjective questionnaire, open space interfaces and high green ratings scenes scored higher in the being away dimension, suggesting that these scenes are better able to provide a differentiation from everyday life and help people escape from stress; lower scores in the extent dimension (implying a higher value of extent) suggest that these scenes have more spatial expanse to attract the users’ attention and provide a chance to take a break. This consistency validates the reliability of assessing the healing properties of streets from different perspectives, suggesting that EEG data and subjective questionnaires can corroborate each other and together reveal the impact of street design on psychological healing.

#### 4.2.2 Complementary perspectives on eye tracking data

Eye-tracking data provides a unique perspective for understanding street healing. Analysis of the heat map of total gaze duration by Gridded AOI revealed that subjects’ gaze duration for areas with medium or high green ratings was significantly longer than for scenes with low green ratings in combination with the street interface, which together with the EEG data and the subjective questionnaire results supported the importance of green ratings for the healing effect. The eye movement data further revealed the distribution of subjects’ visual attention, suggesting that changes in green ratings were more appealing to subjects’ gaze, directly reflecting their attention and interest in natural elements, thus providing a more concrete basis for how to optimize green ratings in street design.

### 4.3 Insights from healing street design practices

Based on the above analysis, this study provides the following insights into street design practice:

(1) Optimize the type of street interface: designers should give priority to open space interfaces with a strong sense of openness and order to avoid excessive visual interference and complexity. A simple spatial layout not only reduces cognitive load, but also enhances an individual’s sense of psychological relaxation by providing a broad field of vision.
(2) Increase the abundance of natural elements: by increasing green ratings, designers can significantly enhance the healing qualities of streets. Natural elements not only provide visual aesthetics, but also create a more comfortable and pleasant environment for individuals by improving environmental quality and regulating the microclimate.
(3) Focus on the synergy between spatial layout and natural elements: the synergy between open space interface and high green ratings has a significant effect on healing. Designers can find the balance point between visual attraction and cognitive load by reasonably matching open space and natural elements, thus maximizing the healing effect.
(4) Multi-dimensional assessment of design effects: through a multi-modal assessment framework that integrates EEG, eye tracking and subjective questionnaires, designers can more comprehensively understand the psychological and physiological impacts of street design on individuals, so as to optimize the design solutions and enhance the healing properties of streets.

## 5 Conclusion

Streets serve as a key link between different areas; they are not only a conduit for transportation, but also an important part of everyday life. However, as the world moves at a rapid pace, street design has tended to overemphasize the transportation function at the expense of its potential to provide spiritual solace. As a result, the concept of healing streets has emerged, which provide a stress-reducing and relaxing environment for residents through the introduction of natural landscapes and well-designed public spaces. Such a street design goes beyond mere transportation purposes and becomes an important place for residents to seek tranquility in their busy lives. Enhancing the healing nature of streets is therefore crucial to improving the quality of life of residents.

This paper quantifies human emotional states and provides insights into the effects of different street spaces on human healing effects through experimental design and data analysis. The study reveals that EEG data analysis shows that among the street interface variables, the open space interface has the most significant healing, while the solid wall interface has the least healing effect. Meanwhile, high and medium green ratings demonstrated superior healing effects compared to low green ratings. In addition, the study found no significant interaction between street interface and green rating. Specifically, the combination of open space interface and medium green rating produced the best healing effect on the participants. The analysis of the eye movement data further indicated that green rating may have a greater influence on the healing effect than the street interface. In addition, the subjective questionnaire results emphasized that being away and extent in the Perceptual Recovery Scale are two key dimensions in street healing research. However, this study only explored the visual perspective and failed to comprehensively consider other senses such as hearing, smell, and touch, which limits the comprehensiveness of the study to some extent.

Based on the comprehensive analysis of EEG, eye-tracking data and subjective feedback questionnaires, the researchers were able to gain a more precise insight into the subjects’ intrinsic needs and identify which design elements could stimulate a deeper healing response. By adopting a multimodal data fusion analysis strategy, designers were able to deepen their understanding of the user’s sensory experience, thereby optimizing the effectiveness and acceptability of the design solutions and providing empirical support for the construction of healing spaces. Future research should focus on exploring how design elements play a role in the psychological healing process of human beings, using interdisciplinary research methods to promote the sustainable development of public spaces and maximize the well-being of residents.

## Data Availability

The datasets presented in this study can be found in online repositories. The names of the repository/repositories and accession number(s) can be found at: the original dataset from the current study is available at https://115.com/s/swhbimv3h88?password=e2a5&# data connections. The download code is e2a5.
